# Coordinated Function of Cellular DEAD-Box Helicases in Suppression of Viral RNA Recombination and Maintenance of Viral Genome Integrity

**DOI:** 10.1371/journal.ppat.1004680

**Published:** 2015-02-18

**Authors:** Chingkai Chuang, K. Reddisiva Prasanth, Peter D. Nagy

**Affiliations:** Department of Plant Pathology, University of Kentucky, Lexington, Kentucky, United States of America; Agriculture and Agri-Food Canada, CANADA

## Abstract

The intricate interactions between viruses and hosts include an evolutionary arms race and adaptation that is facilitated by the ability of RNA viruses to evolve rapidly due to high frequency mutations and genetic RNA recombination. In this paper, we show evidence that the co-opted cellular DDX3-like Ded1 DEAD-box helicase suppresses tombusviral RNA recombination in yeast model host, and the orthologous RH20 helicase functions in a similar way in plants. *In vitro* replication and recombination assays confirm the direct role of the ATPase function of Ded1p in suppression of viral recombination. We also present data supporting a role for Ded1 in facilitating the switch from minus- to plus-strand synthesis. Interestingly, another co-opted cellular helicase, the eIF4AIII-like AtRH2, enhances TBSV recombination in the absence of Ded1/RH20, suggesting that the coordinated actions of these helicases control viral RNA recombination events. Altogether, these helicases are the first co-opted cellular factors in the viral replicase complex that directly affect viral RNA recombination. Ded1 helicase seems to be a key factor maintaining viral genome integrity by promoting the replication of viral RNAs with correct termini, but inhibiting the replication of defective RNAs lacking correct 5’ end sequences. Altogether, a co-opted cellular DEAD-box helicase facilitates the maintenance of full-length viral genome and suppresses viral recombination, thus limiting the appearance of defective viral RNAs during replication.

## Author Summary

A major force in virus evolution is the ability of viruses to recombine and change their genomes rapidly. Similar to viral replication that greatly depends on subverted cellular proteins, viral genetic recombination is also affected by host factors based on genome-wide screens with tomato bushy stunt virus (TBSV) in yeast model host. However, the roles of host factors in the viral genomic RNA recombination process remain elusive. In this paper, we show evidence, in yeast, plants and *in vitro*, that co-opted cellular helicases by TBSV affect viral recombination through suppressing template-switching and replication of the new recombinant viral RNAs. Based on the presented data, a new concept emerges on the roles of co-opted cellular helicases in maintaining viral genome integrity. Altogether, the hijacked cellular DEAD-box helicases are involved in maintenance of full-length viral RNA genome and suppression of viral RNA recombination, thus blocking the appearance of defective or recombinant viral RNAs during replication.

## Introduction

RNA viruses replicate inside cells and they require many cellular factors to complete their infection cycle. The intricate interactions between viruses and hosts include evolutionary arms race and adaptation that is facilitated by the ability of RNA viruses to evolve rapidly due to high frequency mutations and genetic RNA recombination as well as reassortment of genomic components [1–3]. Interestingly, cellular and environmental factors affect viral RNA recombination, which is a process that joins two or more noncontiguous segments of the same RNA or two separate RNAs together [4,5]. Recombination could alter viral genomes by introducing insertions or duplications, combining new sequences, or leading to deletions or rearrangements. RNA recombination also functions to repair truncated or damaged viral RNA molecules [2,5–7]. Viral RNA recombination can affect virus population dynamics, contribute to virus variability, as well as function in genome repair that maintains the infectivity of RNA viruses [3,4].

**Fig 1:**
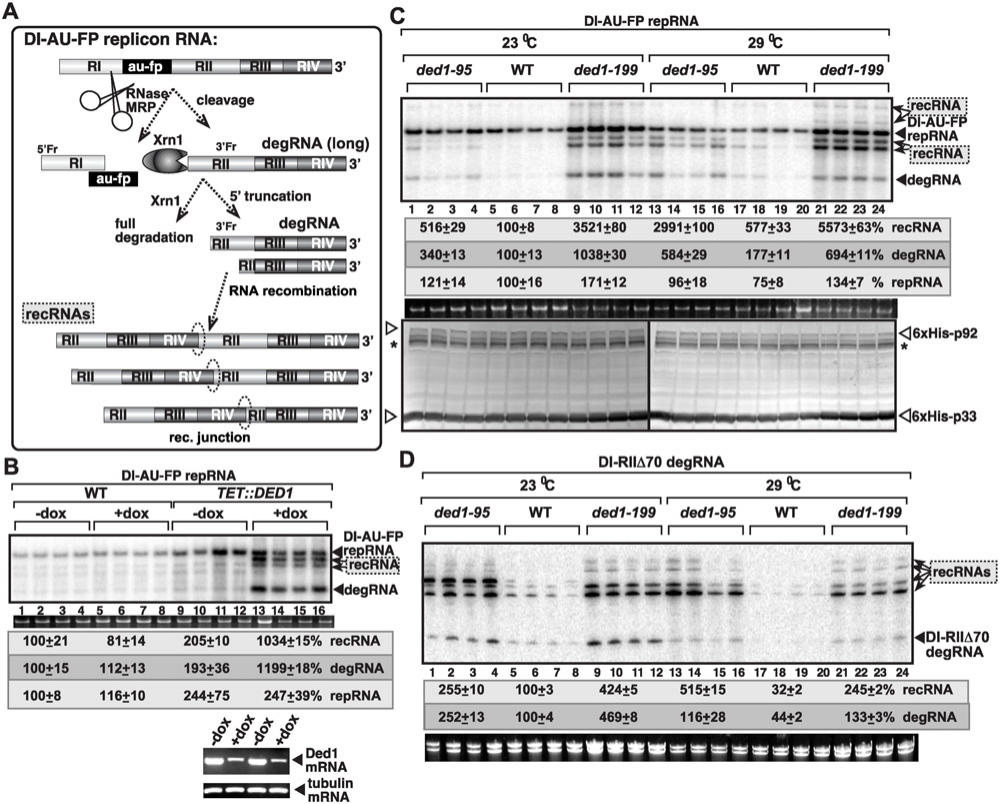
The DDX3-like Ded1p DEAD-box helicase is a suppressor of TBSV RNA recombination. (A) The previously defined viral RNA recombination pathway during TBSV replication. The replication-competent TBSV repRNA is cleaved by cellular endoribonucleases, such as the RNase MRP complex, followed by limited 5’ truncations by the cellular Xrn1p exoribonuclease. These processes lead to the generation of a pool of replication-competent degRNAs that serve as recombinogenic templates in template-switching events driven by the viral replicase. The sequences in recRNAs are shown schematically. (B) Depletion of Ded1p level in yeast leads to the rapid emergence of TBSV recRNAs and degRNAs. Note that doxycycline (+dox samples) leads to depletion of Ded1p expressed from the regulatable *TET* promoter. Replication of the TBSV DI-AU-FP repRNA (see panel A) in wt and TET::DED1 yeasts co-expressing the tombusvirus p33 and p92 replication proteins was measured by Northern blotting 24 h after initiation of TBSV replication. Note the emergence of different species of recRNAs and degRNA (see panel A) in samples with depleted Ded1p. The accumulation level of repRNA was normalized based on the ribosomal (r)RNA (bottom panel). The bottom images show the results with semi-quantitative RT-PCR, which was used to demonstrate knock-down of Ded1 mRNA levels in TET::Ded1 yeast in the presence of doxycycline. Each sample is obtained from independent yeast colonies. The experiments were repeated two-to-three times. Throughout the paper, +/- means standard deviation. (C) Measuring recRNA levels in yeast expressing wt Ded1p, ded1–95^ts^ or ded1–199^ts^ mutants at 23°C (permissive temperature for yeast growth) or 29°C (semi-permissive temperature). Top panel: The accumulation of TBSV DI-AU-FP repRNA, recRNAs and degRNA was measured by Northern blotting at the 24 h time point. Middle panel: The accumulation level of repRNA was normalized based on the ribosomal (r)RNA. Bottom panel: The accumulation levels of His_6_-p92 and His_6_-p33 were tested by Western blotting. Each experiment was repeated. Asterisk marks the SDS-resistant p33 homodimer. (D) Accumulation levels of degRNA and recRNAs in Ded1^ts^ yeasts were measured by Northern blotting. The expressed TBSV template RNA was DI-RIIΔ70 degRNA, which represents a frequently isolated degRNA species lacking RI and part of RII (Panel A). See further details in panel B.

Viral RNA recombination is intensively studied with *Tomato bushy stunt virus* (TBSV), a tombusvirus infecting plants, using yeast (*Saccharomyces cerevisiae*) model host. TBSV is an outstanding model for both replication and recombination studies [8–12]. Systematic genome-wide screens with TBSV have led to the identification of more than 30 host genes affecting viral RNA recombination in yeast [8,9,13–15]. Among the host factors identified is the cytosolic Xrn1p 5’-to-3’ exoribonuclease (Xrn4 in plants) that suppresses TBSV recombination [16–18]. Xrn1p was shown to rapidly degrade cellular endoribonuclease-cleaved TBSV RNAs, termed degRNAs (Fig. 1A) [16–19]. The combined effects of cellular exo- and endoribonucleases determine the accumulation of degRNAs, which are especially active in RNA recombination, and thus, these cellular factors affect the frequency of viral RNA recombination events [9,18]. An additional key cellular factor involved in TBSV recombination is Pmr1 Ca^++^/Mn^++^ pump that controls Mn^++^ level in the cytosol [15]. Studies revealed that the cytosolic Mn^++^ level could greatly affect the properties/activities of the viral replicase, including its ability to synthesize RNA and switch templates. For example, high Mn^++^ level (in the absence of Pmr1) leads to high frequency RNA recombination in yeast or plant cells as well as in a cell-free TBSV replication assay [15].

Tombusviruses code for two replication proteins, termed p33 and p92^pol^, which are translated directly from the genomic (g)RNA. p92^pol^ RNA-dependent RNA polymerase [20,21] is produced through translational readthrough of the p33 stop codon [22–24]. The abundant p33 is an RNA chaperone that functions in RNA template selection/recruitment and in the assembly of the membrane-bound viral replicase complex (VRCs) [21,25–29].

A recent systematic screen with TBSV based on a temperature-sensitive (ts) library of yeast mutants (Prasanth and Nagy, unpublished), identified the yeast Ded1p ATP-dependent DEAD-box RNA helicase as a cellular factor affecting TBSV RNA recombination. Ded1p and ten other yeast DEAD-box proteins, which are the largest family of RNA helicases, were also identified as pro-viral factors in TBSV replication in yeast [13,30–35]. DEAD-box helicases are known to be involved in all aspects of cellular metabolism [36–38], in RNA virus replication [39–42], viral translation [43,44], and affect responses to abiotic stress and pathogen infections [45–47]. They function in RNA duplex unwinding, RNA folding, remodeling of RNA-protein complexes, and RNA clamping [48].

TBSV, which does not code for its own helicase, usurps the yeast DDX3-like Ded1p (similar to the *Arabidopsis* AtRH20 DEAD-box helicase), to promote (+)-strand synthesis [49]. Ded1p/AtRH20 bind to the 3’-end of the TBSV minus-strand RNA, and by locally unwinding the dsRNA replication intermediate structure [50], it renders the promoter sequence accessible to p92^pol^ for initiation of (+)-strand RNA synthesis. Additional DEAD-box helicases, such as Dbp3p (human DDX5-like) and Fal1p (eukaryotic translation initiation factor eIF4AIII-like), which are involved in ribosome biogenesis in yeast [51–53], and the orthologous *Arabidopsis* RH2 and RH5 helicases bind to the 5’ proximal region in the TBSV (-)RNA [54]. This region harbors a critical replication enhancer element (REN) [55]. These co-opted cellular helicases can locally unwind the double-stranded (ds) structure within the REN of the replication intermediate and enhance (+)RNA synthesis [50,54]. Altogether, the co-opted cellular DEAD-box helicases work synergistically to enhance TBSV replication by interacting with the viral (-)RNA, dsRNA and the replication proteins within the VRCs [54].

In this work, we show evidence that Ded1p/AtRH20 helicases are strong suppressors of TBSV recombination in yeast and plants. *In vitro* assays show direct involvement of Ded1p in suppression of viral recombination, which requires its ATPase function. Moreover, the presented data support a new role for Ded1p in facilitating the switch from (-)-strand to (+)-strand synthesis. Interestingly, the eIF4AIII-like AtRH2 helicase enhances TBSV recombination in the absence of Ded1/AtRH20, suggesting that the coordinated action of cellular Ded1/AtRH20 and AtRH2 helicases control viral RNA recombination events. We propose a model on the role of Ded1/AtRH20 in facilitating the replication of full-length viral RNAs with intact 5’ ends while inhibiting the replication of 5’-truncated viral RNAs, thus playing a major role in maintaining the intact genome structure for TBSV.

## Results

### Ded1 mutants support increased level of tombusvirus RNA recombination in yeast

To characterize the role of the DDX3-like Ded1p DEAD-box RNA helicase of yeast in TBSV RNA recombination, first we utilized genetic approaches in yeast. Depletion of Ded1p resulted in ~5-fold increase in TBSV recombinant (rec)RNA accumulation (Fig. 1B, lanes 13–16). Similarly, yeast expressing either Ded1–95^ts^ or Ded1–199^ts^ temperature-sensitive mutants as a single source for Ded1p, led up to 5-to-10-fold increase in TBSV recRNA levels at the semi-permissive temperature (Fig. 1C, lanes 13–16 and 21–24 versus 17–20). Ded1–199^ts^ also supported ~35-fold higher recRNA accumulation at a lower (permissive) temperature (Fig. 1C, lanes 9–12), suggesting that this particular mutant is especially suitable for viral RNA recombination studies. Ded1–199^ts^ (G_368_D mutation) is known to debilitate its function in protein translation and intron splicing [56], while Ded1–95^ts^ (T_408_I mutation) does not affect splicing, but maybe involved in translation and RNA decay [57]. Altogether, the above yeast genetic approaches have conclusively demonstrated that the wt Ded1p helicase is a strong suppressor of TBSV RNA recombination in yeast cells.

The most frequent recombinants in the TBSV system are generated via template-switching mechanism by the viral replicase using viral RNA templates that are cleaved by cellular endo- and exoribonucleases (schematically shown in Fig. 1A) [5,8,9,15,17,18,58]. The partially degraded (5’-truncated) viral RNA products generated by the cellular nucleases are called degRNAs, which serve as templates for viral RNA recombination (Fig. 1A) [8,9]. Interestingly, the amounts of degRNAs also increased by ~3-to-10-fold, suggesting their efficient generation and replication in Ded1^ts^ mutant yeasts (Fig. 1C) or in yeast with depleted Ded1p (Fig. 1B). Interestingly, the degRNAs are superior templates for high frequency recombination when expressed in yeast cells in the presence of the viral p33/p92^pol^ replication proteins (Fig. 1D) [8,9]. Both Ded1–95^ts^ and Ded1–199^ts^ supported 3-to-8-fold higher recRNA accumulation from the DI-RIIΔ70 degRNA template than the wt Ded1p did in yeast (Fig. 1D). These data further supported the suppressor function of Ded1p in TBSV recRNA accumulation in yeast.

The surprisingly robust accumulation of the 5’-truncated degRNAs in both ded1–95^ts^ and ded1–199^ts^ yeasts expressing the full-length DI-AU-FP repRNA (Fig. 1C) was likely due to enhanced efficiency of their replication, because expression of the representative DI-RIIΔ70 degRNA accumulated to high level (up to ~5-fold increase) in ded1–95^ts^ and ded1–199^ts^ yeast strains in comparison with the wt yeast (Fig. 1D). These findings indicate an unexpected role of Ded1p in suppressing the replication of the 5’-truncated degRNAs. This is in contrast with the pro-viral role of Ded1p in enhancing the accumulation of TBSV DI-72 repRNA, which carries the authentic 5’ end sequence (see also below) [49,54].

### Ded1 mutants support increased level of (-)RNA products from TBSV recRNAs and degRNAs in yeast

**Fig 2:**
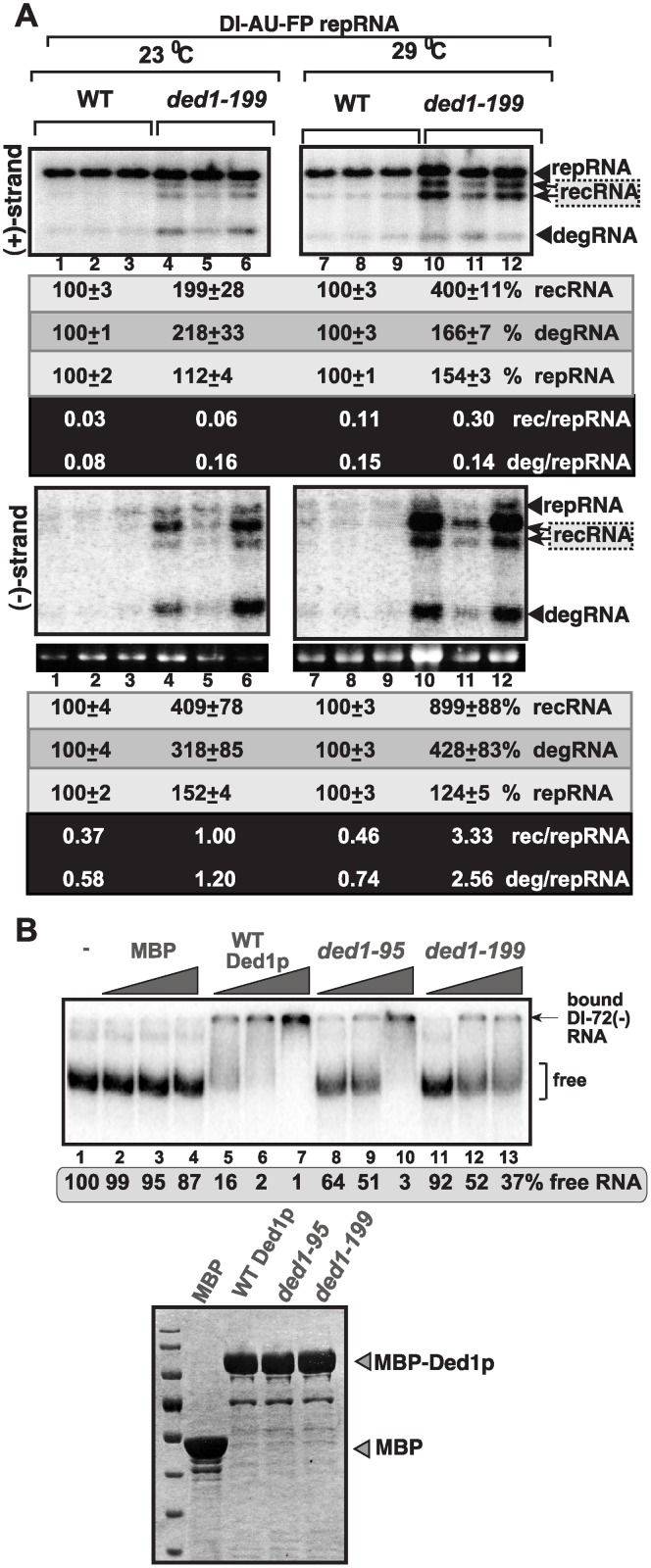
Increased level of TBSV minus-strand synthesis on recRNAs and degRNAs in yeast expressing ded1–199^ts^ mutant. (A) Top and bottom panels: Northern blot analysis of (+) and (-)RNA levels, respectively, in wt and ded1–199^ts^ yeast.The ratios of the most abundant recRNAs versus repRNA (DI-AU-FP) and degRNA versus repRNA were calculated. See further details in Fig. 1. Note that viral RNA recombination is a chance event depending on many factors and when the first recombination event occurs, thus influencing the outcome. Quantitation is based on multiple repeats (different yeast streaks) in each experiment, and despite the different numbers on recRNAs, both Figure 1 and 2 show the same trend. (B) Top panel: *In vitro* RNA binding assay with purified wt Ded1p and mutants. The assay contained the ^32^P-labeled DI-72 (-)repRNA (~0.1 pmol) plus increasing amount of MBP, MBP-Ded1, MBP-Ded1–95 or MBP-Ded1–199 (all were used in 40, 80 and 160 nM). The free or Ded1p-bound ssRNA was separated on nondenaturing 5% acrylamide gels. Bottom panel: The MBP-affinity purified MBP-Ded1 and mutants are analyzed on a SDS-PAGE gel.

Testing the accumulation of (+) versus the (-)RNA products revealed ~9-fold increased (-)recRNA production in case of ded1–199^ts^ yeast at semi-permissive temperature in comparison with wt yeast (Fig. 2A). Interestingly, similar to the high level of (-)recRNAs, accumulation of truncated (-)degRNAs was also increased by ~4-fold, while the amount of full-length (-)repRNA changed only slightly (DI-AU-FP repRNA, Fig. 2A) in ded1–199^ts^ yeast. Altogether, (-)recRNAs accumulated to ~3-fold higher level than the full-length DI-AU-FP (-)repRNA in ded1–199^ts^ yeast (Fig. 2A). On the contrary, the DI-AU-FP repRNA was the most prevalent (+)RNA product, while the (+)recRNAs and (+)degRNA products accumulated to 3-to-7-fold lesser amounts than the (+)repRNA in ded1–199^ts^ yeast (Fig. 2A).

Since ded1–95^ts^ and ded1–199^ts^ mutations are present within the RNA binding domain of the Ded1p helicase [56], we have tested if the mutants show altered viral RNA binding characteristic when compared with the wt Ded1p. The EMSA assay with DI-72(-) RNA template revealed that the purified ded1–95^ts^ and ded1–199^ts^ mutants bound to the viral (-)RNA with up to 25-fold reduced efficiency *in vitro* (Fig. 2B). The low efficiency in viral (-)RNA binding by these Ded1p mutants could be the reason for these mutants supporting the increased rate of viral recombination, high level of degRNA accumulation and reduction in viral (+)-strand synthesis (see Discussion).

**Fig 3:**
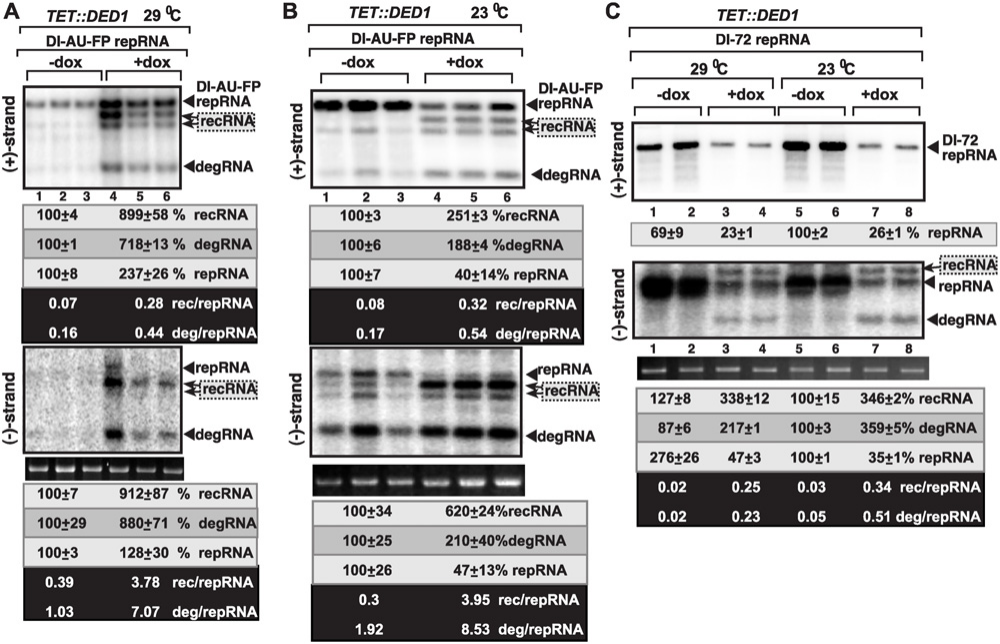
Ded1p depletion leads to increased level of TBSV minus-strand synthesis on recRNAs and degRNAs in yeast. (A) Top and bottom panels: Northern blot analysis of (+) and (-)RNA levels, respectively, in TET::DED1 yeast after depletion of Ded1p level (+dox samples) or normal Ded1p level (-dox, no doxycyclin added).The ratios of the most abundant recRNAs versus repRNA (DI-AU-FP) and degRNA versus repRNA were calculated. See further details in Fig. 1. (B) Similar to panel A, except TET::DED1 yeast was grown at 23°C. (C) Similar to panel A, except DI-72 repRNA was expressed in TET::DED1 yeast for TBSV recombination studies. Note that DI-72 repRNA is a much better template than the longer DI-AU-FP repRNA. Also, the recRNAs are larger than DI-72 repRNA, as indicated by arrowheads.

In comparison with the results obtained via ded1–95^ts^ and ded1–199^ts^ mutants, we observed a similar trend with increased accumulation of (-)recRNA and (-)degRNA products obtained with the recombinogenic DI-AU-FP repRNA, when yeast expressed Ded1p at a reduced level (+doxycycline treatment, Fig. 3A-B). Altogether, these data revealed that Ded1p is important in regulation of (+) versus (-)RNA products and this regulation depends on the presence of the authentic 5’ end sequence from TBSV (+)repRNA.

To confirm the importance of co-opted Ded1p in viral RNA replication and recombination, we also tested the accumulation of various Δ+) and (-)RNA products with the efficient DI-72 repRNA, which replicates to the highest level among all TBSV RNAs in yeast and plants cells [59,60]. As expected based on previous publications [49,61], depletion of Ded1p by doxycycline in *TET*::*DED1* yeast, reduced the accumulation of DI-72 (+)repRNAs by ~4-fold, while the accumulation of new (+)recRNAs and (+)degRNA products was below the detection limit (top image in Fig. 3C, lanes 3–4 and 7–8). Interestingly, however, (-)recRNA and (-)degRNA products, which were almost as abundant as the full-length DI-72 (-)repRNA, were detected in yeasts with depleted Ded1p level (bottom image in Fig. 3C, lanes 3–4 and 7–8). The corresponding (-)recRNA and (-)degRNA products were below detection limit in yeasts expressing Ded1p to high level (bottom image in Fig. 3C, lanes 1–2 and 5–6). Altogether, these results demonstrate that Ded1p plays a critical role in suppression of the formation and accumulation of recRNA and degRNA products during minus-strand synthesis.

### Ded1 suppresses the formation of recRNAs and the replication of the 5’-truncated degRNAs *in vitro*

**Fig 4:**
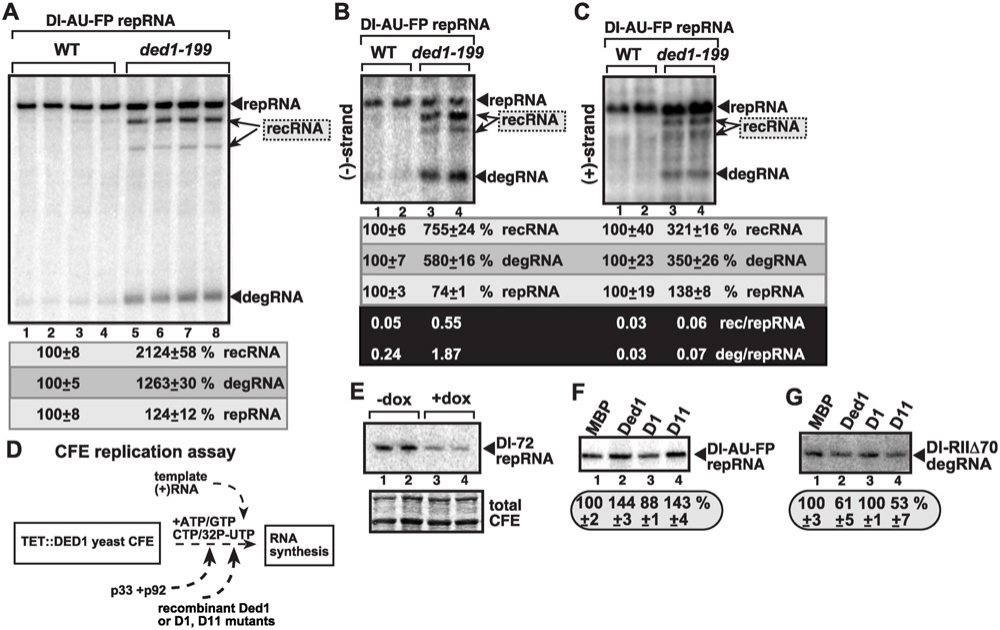
Suppression of TBSV recRNA accumulation by Ded1p in *in vitro* replication assays. (A) The membrane-enriched fraction (MEF) was isolated from wt or ded1–199^ts^ yeast expressing the tombusvirus p33 and p92 replication proteins in combination with the DI-AU-FP repRNA, followed by *in vitro* tombusvirus replication assay. The denaturing PAGE analysis shows the emergence of recRNAs and degRNA as indicated. (B–C) Northern blot analysis of the polarity of the TBSV RNAs synthesized in the MEF assay (see panel A, except the assay was done with nonlabeled ribonucleotides) using ^32^P-labeled probes to detect (-)-strands and (+)-strands, respectively. The ratios between various TBSV RNAs were calculated as in Fig. 2. (D) Scheme of the yeast cell-free (CFE) assay for TBSV replication. Note that the assembly of the TBSV replicase takes place in the CFE using recombinant viral components as shown. The TBSV p33 and p92 replication proteins and Ded1p and its mutants (D1 is inactive, D11 has enhanced ATPase activity) were purified from *E*. *coli*. (E) Reduced replication of the full-length DI-72 repRNA in TET::DED1 yeast CFEs with high or depleted levels of Ded1p (see Fig. 1). (F-G) Denaturing PAGE analysis of the effect of Ded1p and its mutants on the replication of the full-length recombinogenic DI-AU-FP and the 5’-truncated DI-RIIΔ70 degRNA, respectively, in the CFE assay. The experiments were repeated twice.

To dissect the inhibitory function of Ded1p in recRNA formation and degRNA replication, first we used an *in vitro* assay with isolated yeast membranes [62]. The yeast membrane fraction contains the tombusvirus replicase in complex with the viral RNAs, thus facilitating studies on the viral RNAs functionally associated with the replicase. Denaturing PAGE analysis of the *in vitro* replicase products revealed that both recRNAs and degRNAs were actively replicated by the tombusvirus replicase derived from ded1–199^ts^ yeast (~13-to-21-fold higher level than in wt replicase), while these RNAs were barely detectable in the replicase from wt yeast (Fig. 4A). In addition, ded1–199^ts^ replicase supported ~6-to-7-fold higher level of (-)recRNAs and (-)degRNAs in comparison with slightly reduced DI-AU-FP (-)repRNA carrying the authentic 5’ end sequence *in vitro* (Fig. 4B). The (+)recRNAs and (+)degRNAs accumulated to 3-fold higher level in ded1–199^ts^ yeast than the corresponding RNAs in wt yeast, but (+)recRNAs and (+)degRNAs were ~12-fold less abundant than the DI-AU-FP (+)repRNA *in vitro* (Fig. 4B-C). Thus, similar to the situation in yeast cells, wt Ded1p suppressed *in vitro* (-)-strand synthesis with the recRNAs and degRNAs, but not with DI-AU-FP repRNA carrying the authentic 5’ end sequence.

The second assay was based on a cell-free extract (CFE) from yeast with depleted Ded1p that was used to assemble the tombusvirus replicase *in vitro* using purified recombinant p33/p92^pol^ and (+)repRNAs (Fig. 4D) [49]. The CFE supports a complete replication cycle resulting in both (-) and (+)-stranded repRNA products [29]. As expected, Ded1p facilitates the production of (+)repRNAs carrying the authentic 5’ end sequence [Fig. 4E, lanes 3–4, see reduced DI-72 repRNA accumulation in CFE with depleted Ded1p (+dox)] [49]. Similarly, addition of the purified recombinant wt Ded1p to the CFE programmed with DI-AU-FP (+)repRNA, which carries the authentic 5’ end sequence, led to a ~50% increase in repRNA accumulation (Fig. 4F, lane 2), while the ATPase-deficient D1 mutant of Ded1p [49,63] could not stimulate repRNA replication *in vitro* (Fig. 4F, lane 3). On the contrary, addition of the purified recombinant wt Ded1p or D11 mutant with increased ATPase activity [63] to the CFE with the 5’-truncated DI-RIIΔ70 degRNA, led to ~40–50% decrease in degRNA accumulation (Fig. 4G, lanes 2 and 4 versus lane 1), while D1 mutant did not affect the replication of DI-RIIΔ70 degRNA in the CFE (lane 3). Based on these data from CFE assays, we conclude that Ded1p inhibits the replication of recRNAs or degRNAs missing the authentic 5’ end sequence likely through blocking the (-)-strand synthesis on these RNA templates.

### Ded1p helicase promotes the release of the p92 RdRp protein from the template RNA *in vitro*

**Fig 5:**
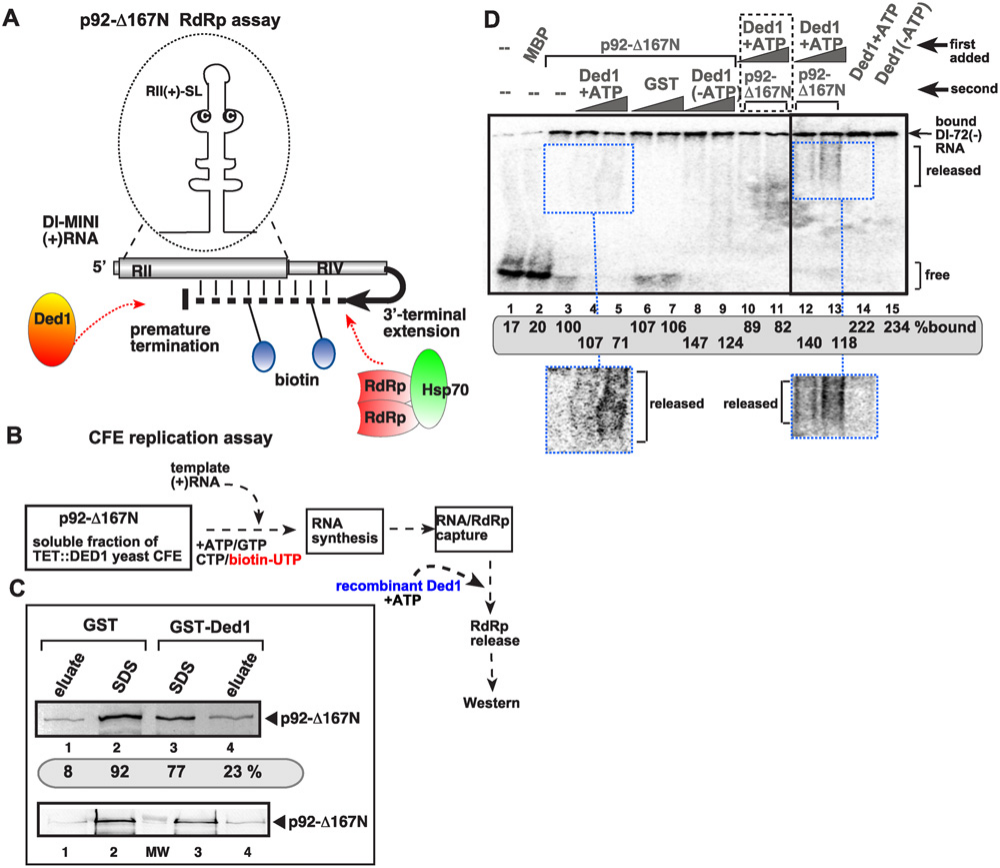
Ded1p DEAD-box helicase promotes the release of the viral RNA from the p92 RdRp *in vitro*. (A) Scheme of the *in vitro* assay to produce biotinylated RNA bound to TBSV RdRp. We used an *in vitro* activated purified p92 derivative (called p92-Δ167N), which can specifically use TBSV-derived (+)RNA template for RNA synthesis *in vitro* for 3’terminal extension on the added template RNA as shown schematically [21]. (B) The scheme shows the *in vitro* RdRp reaction consisting of p92-Δ167N RdRp and soluble fraction of yeast CFE (with depleted endogenous Ded1p due to the use of TET::Ded1 yeast) to activate the RdRp function. The activated RdRp then introduces biotin-labeled UTP to the template, allowing for the capture of the RNA product (a hairpin-like dsRNA) associated with p92-Δ167N RdRp on streptavidin-coated agarose beads. Then the addition of purified Ded1p might facilitate the release of the p92-Δ167N RdRp from the RNA. (C) The amounts of eluted versus bound p92-Δ167N RdRp was measured by Western blotting (see images). The eluate contains the released p92-Δ167N RdRp from the streptavidin beads, while the SDS fraction contains p92-Δ167N RdRp that was left on the beads after Ded1p treatment. (D) EMSA assay with p92-Δ167N RdRp in the presence of Ded1p helicase. The ^32^P-labeled RNA probe was RI(+), which is bound by both p92-Δ167N RdRp and Ded1p *in vitro*. The RNA probe and the first added protein (indicated at the top) was allowed to form an RNP complex, followed by the addition of the second protein 15 min latter. We used 0.4 μg of affinity-purified MBP-p92-Δ167N RdRp and 1.0 and 2.0 μg of affinity-purified GST-Ded1p or GST. Lanes 10–11 contain samples with both proteins added to the ^32^P-labeled RNA probe at the same time. The bound ^32^P-labeled RNA probe was quantitated. Note that the diffused band (“smear”) likely represent the released RNA probe from the RNP complex (see the enhanced images at the bottom) only under the given conditions. The higher values with Ded1p (lanes 14–15) are likely due to the better RNA-binding by Ded1p then p92-Δ167N (lane 3).

Based on known features of DEAD-box helicases in remodeling protein-RNA complexes [48,64], we reasoned that Ded1p might be involved in releasing the p92 RdRp protein from the (+)RNA template at the end of (-)-strand synthesis, thus decreasing the chance for template-switching events (see Discussion). To test this model, we developed an *in vitro* assay with a soluble form of p92, called p92-Δ167N, which can specifically use TBSV-derived (+)RNA template for RNA synthesis *in vitro* in the presence of biotynylated UTP and other ribonucleotides as shown schematically in Fig. 5A [21]. The biotynylated viral dsRNA form was then captured via streptavidin beads (Fig. 5B). We then added purified Ded1p to the beads to facilitate the putative release of the p92-Δ167N RdRp from the captured dsRNA product. The amount of dsRNA-bound versus released p92-Δ167N was measured by Western blotting (Fig. 5B-C). These experiments revealed that three-times more p92-Δ167N was released from the viral dsRNA product when purified wt Ded1p was included in the assay (Fig. 5C, lanes 4 versus 1).

In another assay, we used EMSA with MBP-p92-Δ167N and purified GST-Ded1p based on ^32^P-labeled RI(+) RNA as a probe. Both MBP-p92-Δ167N and GST-Ded1p bind to the probe when applied alone (Fig. 5D, lanes 3 and 14, respectively). However, when we added p92-Δ167N first to the probe, followed 15 min latter by addition of GST-Ded1p, then the release of the probe was detectable in the form of diffused label (“smear”) (Fig. 5D, lanes 4–5 versus 6–7 with purified GST as a control). Interestingly, the release of the probe was dependent on the presence of ATP, suggesting that Ded1p requires ATP for this function (Fig. 5D, lanes 8–9 versus 4–5). The diffused label was also observed when Ded1p was added first to the RNA, followed by p92-Δ167N (Fig. 5D, lanes 12–13), suggesting that Ded1p and p92-Δ167N likely form a complex that releases the viral RNA.

### Ded1 suppresses recombination and the replication of the 5’-truncated degRNAs independent of Xrn1p 5’-to-3’ exoribonuclease in yeast

**Fig 6:**
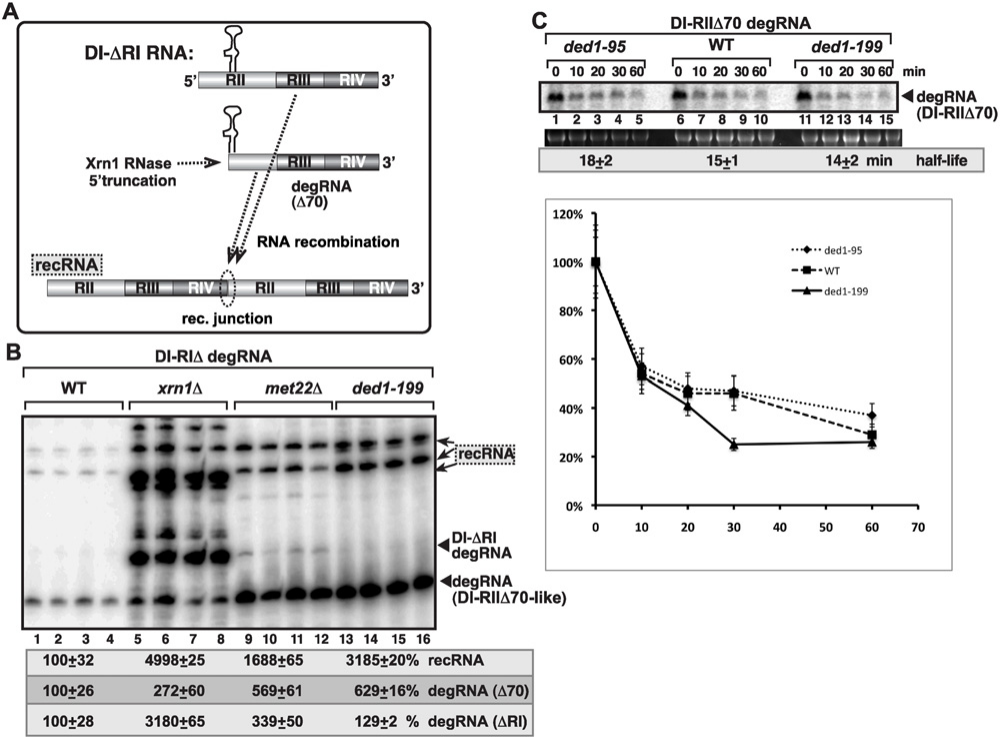
Suppression of viral RNA recombination by Ded1 helicase is independent of the Xrn1 pathway. (A) Schematic representation of the previously characterized Xrn1-driven TBSV RNA recombination pathway in yeast. Plasmid-driven expression of the DI-(RI in the presence of p33/p92 replication proteins leads to partial 5’ truncations by the cellular Xrn1p 5’-to-3’ exoribonuclease generating DI-RIIΔ70-like degRNAs as shown. DI-RIIΔ70-like degRNAs then participate in RNA recombination as shown. (B) Recombination profile of DI-(RI RNA in *xrn1Δ*, *met22Δ* (as a control) and in ded1–199 yeasts. The original expressed DI-(RI degRNA, DI-RIIΔ70-like degRNAs and recRNAs are depicted with arrowheads and arrows, respectively. Note that the recRNA profile is dramatically different in *xrn1Δ* yeast when compared to ded1–199 yeast. (C) Half-like of DI-RIIΔ70 degRNA in WT, ded1–95 and ded1–199 yeasts. Note that yeast did not express p33/p92 proteins.

To establish the function of Ded1p during TBSV replication and RNA recombination, we examined if Ded1p affects these processes via controlling Xrn1p 5’-to-3’ exoribonuclease, which is a key enzyme in TBSV RNA stability and for suppression of TBSV RNA recombination in yeast [5,16–18,65]. For these studies, we expressed a 5’-truncated repRNA (DI-ΔRI, Fig. 6A), which goes through further 5’-truncations (up to ~70 nt, where RII(+)-SL hairpin structure stops the nuclease activity) in the presence of Xrn1p in wt yeast (Fig. 6A), while this truncation process is weak in *xrn1Δ* yeast (Fig. 6B) [65]. DI-ΔRI RNA did not accumulate in ded1–199^ts^ yeast, similar to wt yeast (Fig. 6B, lanes 13–16 and 1–4), while DI-(RI accumulated to high level in *xrn1Δ* yeast (Fig. 6B, lanes 5–8). Also, the profile of recRNAs accumulating in ded1–199^ts^ yeast was similar to that in wt yeast and different from that in *xrn1Δ* yeast (Fig. 6B). Thus, it seems that ded1p mutation does not affect TBSV RNA recombination and degRNA accumulation via inhibition of the Xrn1p activity. This conclusion was further supported by RNA stability experiments that showed comparable half-life for degRNA in ded1–95^ts^ and ded1–199^ts^ yeasts to the wt yeast (Fig. 6C).

### AtRH2 DEAD-box helicase promotes the formation of recRNAs and the replication of the 5’-truncated degRNAs in yeast

**Fig 7:**
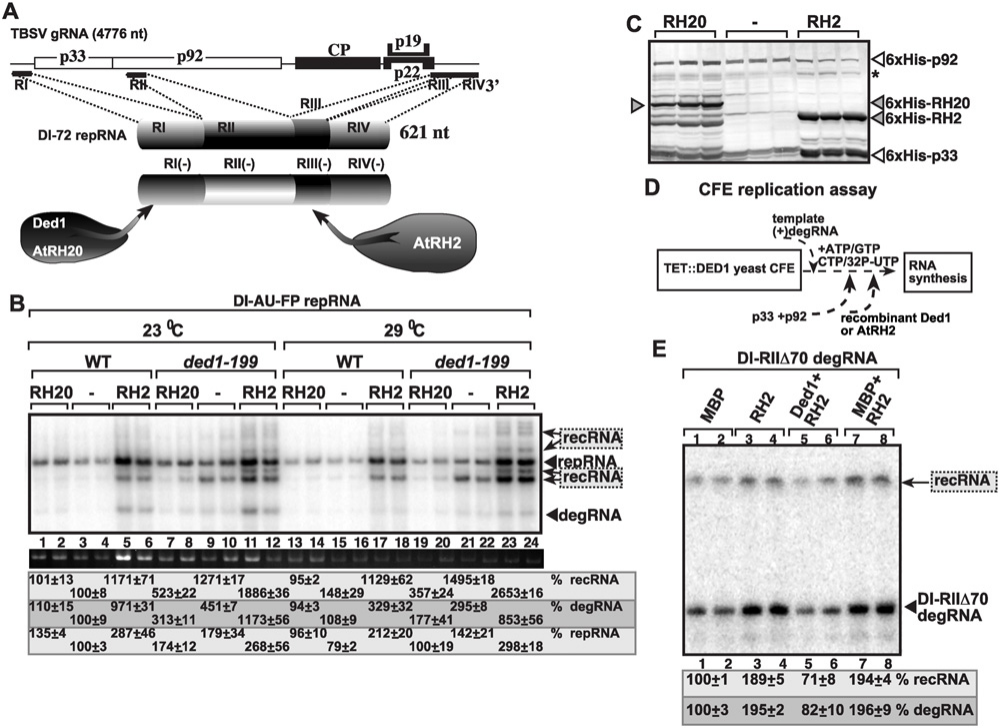
Opposite effects of subverted cellular DEAD-box helicases on TBSV recombination in yeast and in the CFE assay. (A) TBSV co-opt two groups of cellular helicases that become part of the VRCs: the DDX3-like Ded1/AtRH20 and the eIF4AIII-like AtRH2. These helicases bind to different *cis*-acting replication elements present at the 3’ and 5’ ends of the viral (-)RNA as shown. Note that RI(-) carries the promoter for (+)-strand synthesis, while the RIII(-) is a replication enhancer element (REN). (B) Over-expression of AtRH2 enhances recRNAs and degRNA accumulation, while over-expression of AtRH20 decreases the occurrence of these RNAs in wt or ded1–199^ts^ yeasts. The Northern blot analysis was done as in Fig. 1. (C) Western blot analysis to detect the expression level of His_6_-tagged cellular helicases and the His_6_-tagged viral replication proteins in the WT and ded1–199^ts^ yeasts. Asterisk marks the SDS-resistant p33 dimer. (D) The scheme of the CFE-based TBSV replication assay. Note that the level of Ded1p was depleted in TET::DED1 yeast prior to CFE isolation. (E) Denaturing PAGE analysis of the accumulation of 5’-truncated DI-RIIΔ70 degRNA used as the original template in the CFE assay and the *in vitro* generated recRNA. See further details in Fig. 3.

Because TBSV replication is known to depend on two types of cellular DEAD-box helicases, namely the DDX3-like Ded1p/AtRH20 that bind to the 3’end of the (-)RNA and the eIF4AIII-like Fal1p/AtRH2 helicases that bind to a 5’ proximal enhancer element in the (-)RNA (Fig. 7A) [49,54], we also tested the effect of expression of AtRH2 on TBSV recombination in yeast. We observed up to ~12-fold enhanced level of TBSV RNA recombination in wt and 26-fold increase in ded1–199^ts^ yeasts expressing AtRH2 (Fig. 7B, lanes 5–6, 17–18 and 11–12, 23–24). In contrast, expression of AtRH20 helicase (Fig. 7C), which is a Ded1p ortholog, suppressed recRNA accumulation in both wt and ded1–199^ts^ yeasts (Fig. 7B). Thus, different co-opted cellular helicases have opposite effects on TBSV recombination in yeast.

To test if AtRH2 has direct function in TBSV recombination, we used the CFE-based TBSV replication assay prepared from yeast with depleted Ded1p (Fig. 7D). Interestingly, the addition of purified recombinant AtRH2 increased the replication of the 5’-truncated DI-RIIΔ70 degRNA by ~2-fold and RNA recombination also by ~2-fold (Fig. 7E, lanes 3–4 versus 1–2). However, the stimulatory effect of AtRH2 on RNA recombination is neutralized by the addition of purified recombinant Ded1p helicase (Fig. 7E, lanes 5–6), suggesting that AtRH2 only promotes formation of recRNAs and the replication of the 5’-truncated degRNAs when Ded1p helicase is depleted. In other words, Ded1p helicase seems to be the dominant factor with its recombination suppressor activity.

### Opposite roles of AtRH2 and AtRH20 plant helicases in viral RNA recombination and the replication of the 5’-truncated degRNAs in plants

**Fig 8:**
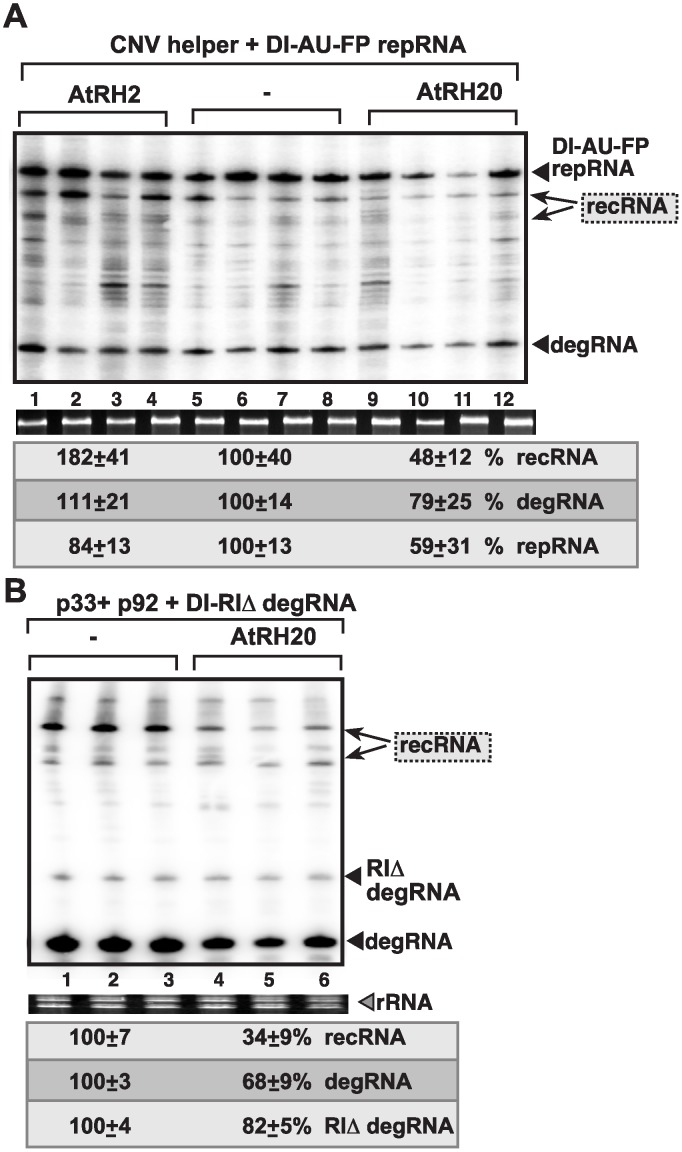
Opposite effects of subverted cellular DEAD-box helicases on TBSV recombination in plants. (A) Over-expression of AtRH2 or AtRH20 was done in *N*. *benthamiana* leaves by agroinfiltration. The same leaves were agro-infiltrated to co-express CNV helper virus and the DI-AU-FP repRNA from the 35S promoter. The control samples were obtained from leaves not expressing AtRH2 or AtRH20 proteins (lanes 5–8). Total RNA was extracted from leaves 4 days after agroinfiltration. The accumulation of repRNA, recRNAs and degRNA in *N*. *benthamiana* leaves was measured by Northern blotting (top panel), see Fig. 1 for details. The ribosomal RNA (rRNA) was used as a loading control and shown in agarose gel stained with ethidium-bromide (bottom panel). (B) Over-expression of AtRH20 inhibits recRNA formation from the degRNAs. AtRH20 was over-expressed in *N*. *benthamiana* leaves by agroinfiltration. The same leaves were co-agro-infiltrated to co-express CNV p33 and p92 replication proteins and the DI-RIΔ degRNA from the 35S promoter. The control samples were obtained from leaves not expressing AtRH20 protein (lanes 1–3). See further details in panel A.

To confirm the roles of the above cellular helicases in TBSV RNA recombination in plants, we over-expressed AtRH2 and AtRH20 in *Nicotiana benthamiana* plants also expressing DI-AU-FP repRNA in the presence of *Cucumber necrosis virus* (CNV), a closely related tombusvirus that serves as a helper virus for the TBSV repRNA. The helper tombusvirus provides the p33 and p92 replication proteins *in trans* for the replication of repRNA and the *de novo* generated recRNAs in this system. We found that the Ded1p ortholog AtRH20 suppressed TBSV recRNA accumulation by ~2-fold, while AtRH2 increased recRNAs by ~2-fold (Fig. 8A). These data indicate that the different co-opted cellular helicases have opposite effects on TBSV recombination in plants. Over-expression of AtRH20 also suppressed the accumulation of the 5’-truncated DI-RIΔ degRNA and the further truncated degRNAs, ultimately resulting in ~3-fold less recRNA accumulation than in control plants (Fig. 8B, lanes 4–6 versus 1–3). Based on these results, we suggest that the roles of the two cellular helicases in plants are comparable to the functions of these helicases *in vitro* in the CFE assay and in yeast.

## Discussion

Viral RNA recombination and the generation of defective viral RNA molecules are thought to be chance events that take place during viral RNA replication. It is possible that RNA viruses regulate these unique processes to guarantee the efficient replication of the full-length viral RNA and to reduce the competition of various viral RNAs for viral- and host factors. Accordingly, the role of viral replicase proteins in viral RNA recombination and defective RNA generation has been documented before [4,66–71]. However, based on systematic genome-wide screens performed with TBSV in yeast surrogate host [8,9], a new concept on the key roles of cellular factors in viral RNA recombination and defective RNA generation is emerging [5,15–18,58,65]. Among such cellular factors are DEAD-box helicases as demonstrated in this paper.

### DDX3-like Ded1 DEAD-box helicase is a strong suppressor of tombusvirus RNA recombination in yeast

This work based on genetic approaches with Ded1p ts mutants or depletion of Ded1p in yeast and *in vitro* approaches with cell-free replication of TBSV RNAs strongly supports a TBSV recombination suppressor activity of the co-opted Ded1p cellular helicase. Since the ATPase-deficient D1 mutant of Ded1p does not have recombination suppressor activity *in vitro* (Fig. 4), it seems that Ded1p helicase has a direct inhibitory function in TBSV RNA recombination. The AtRH20 helicase, a plant ortholog of Ded1p, also has similar recombination suppressor activity in yeast and in plants. Importantly, the recombination suppressor activity of Ded1p is independent of the recombination suppressor activity of the previously characterized Xrn1p 5’-to-3’ exoribonuclease, which acts by efficiently removing degRNAs and recRNAs generated during TBSV replication (Fig. 6) [16–18,65]. Altogether, Ded1p helicase is the first co-opted cellular factor in the viral replicase complex that has been shown to directly affect viral (+)RNA recombination.

### A novel role of Ded1 in maintaining genome integrity and in suppression of the replication of recRNAs and the 5’-truncated degRNAs

**Fig 9:**
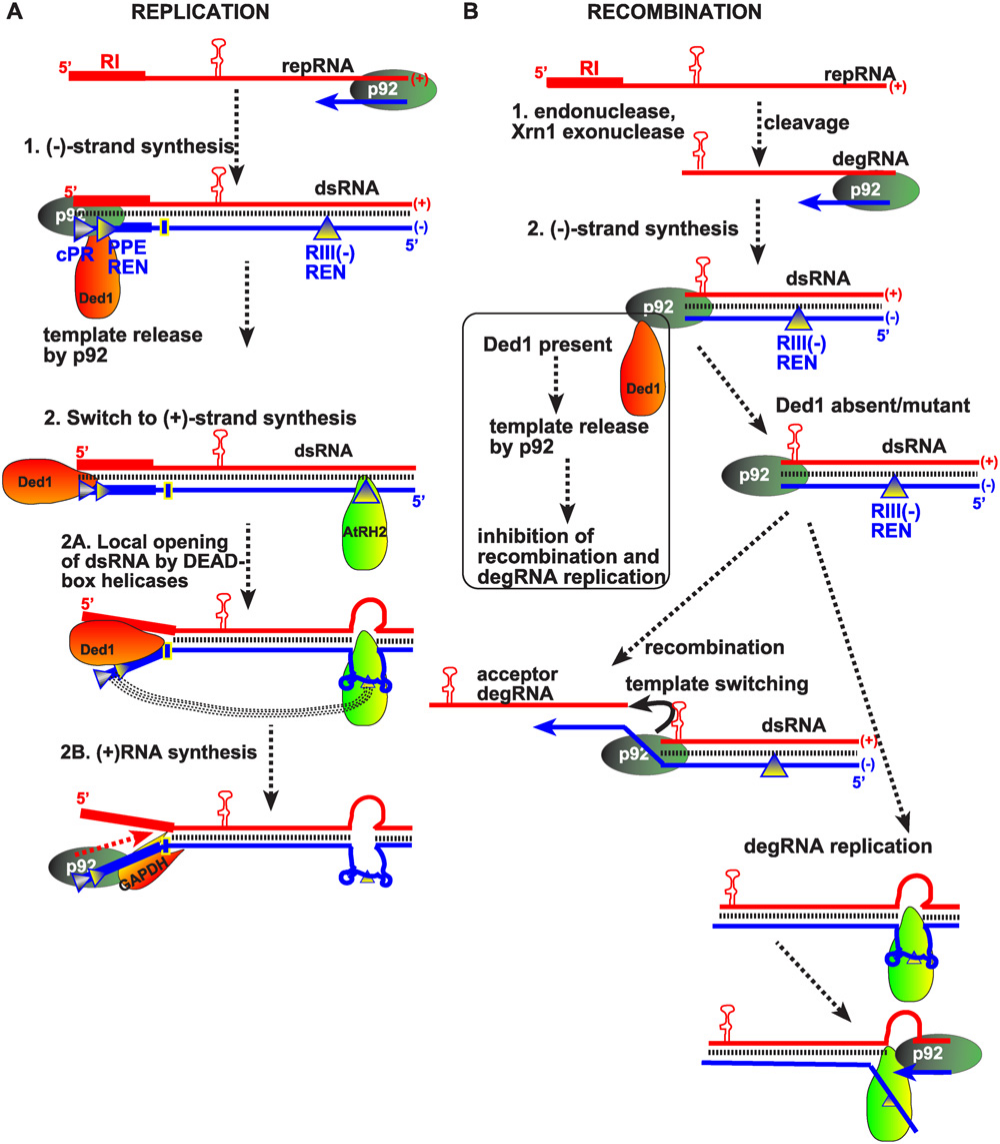
Models showing the functions of subverted cellular DEAD-box helicases in TBSV replication and viral RNA recombination. (A) We propose that the DDX3-like Ded1/AtRH20 helicase facilitates the release of the pausing viral RdRp (shown as p92) at the end of the template, leading to switch from (-)-strand synthesis to (+)-strand synthesis. The previously defined additional functions of Ded1p and the eIF4AIII-like AtRH2 helicase is to open the dsRNA at the promoter region (cPR plus promoter proximal replication enhancer, PPE REN, indicated by rectangles) and the RIII(-)replication enhancer, as shown schematically [49,54,61]. Also shown is the long-range RNA-RNA interaction between the RIII(-) REN and the cPR region, which is proposed to help repeated use of the (-)RNA of the dsRNA as template for (+)RNA synthesis. The hairpin structure indicates the critical RII(+)-SL sequence required for the p33 replication protein-driven recruitment of the viral (+)RNA into replication. The RI(+) and the complementary RI(-) RNA sequences bound by Ded1p are shown with wide bars. (B) Our model suggests that Ded1/AtRH20 suppresses viral RNA recombination by facilitating the release of the pausing p92 at the end of degRNA recombinogenic template (created by cellular nucleases as shown). This activity of Ded1p results in suppression of RNA recombination (template-switching by p92), see the boxed portion of the model. Depletion of Ded1p or when Ded1p is mutated (ded1–199^ts^), p92 could stay on the degRNA template that likely promotes template-switching to a new (+)degRNA as shown as acceptor degRNA or to the 3’ end of the same (+)degRNA. On the other hand, in the absence of Ded1p, the eIF4AIII-like AtRH2 helicase could facilitate (-)RNA synthesis by opening up the dsRNA intermediate of degRNA that might lead to re-loading p92 RdRp to the 3’end of the (+)-strand for a new round of (-)degRNA synthesis. Similar activity by AtRH2 on degRNA might also facilitate template-switching by p92 RdRp for more efficient recombination. Altogether, these models take into account the co-ordinated actions of the two groups of subverted cellular helicases during replication and recombination.

A previously demonstrated function of co-opted Ded1p helicase is to locally unwind the double-stranded RNA replication product after the (-) RNA synthesis is completed on the (+)RNA template (Fig. 9A) [49,50,54]. Ded1p then facilitates the loading of the viral replicase onto the 3’ end of the (-)-stranded RNA portion of the dsRNA intermediate with the assistance of the co-opted cellular glyceraldehyde-3-phosphate dehydrogenase (GAPDH) [49,50,54]. Thus, ultimately, Ded1p promotes the asymmetrical (i.e., excess) production of new (+)-strand RNAs by allowing the selective use of the (-)RNA in the dsRNA intermediate template.

However, this work also reveals a new role of Ded1p in inhibition of (-)-strand synthesis, especially with those RNA templates that lack authentic 5’ sequences, such as degRNAs and recRNAs (Figs. 2–3). Interestingly, the amount of (-)recRNAs and (-)degRNAs far exceeds the DI-AU-FP (-)repRNA in Ded1p deficient yeast or *in vitro* when Ded1p mutant is present, while the (+)repRNA is more abundant than (+)recRNAs or (+)degRNAs (Figs. 2–3). In case of the highly efficient DI-72 repRNA, the (-)recRNAs and (-)degRNAs are only detected when Ded1p is depleted (Fig. 3). Thus, one major function of the co-opted wt Ded1p is to promote the efficient replication of only the full-length viral RNAs, while suppressing the replication of 5’-truncated viral RNAs, lacking critical *cis*-acting elements. This novel function of Ded1p in maintenance of genome integrity seems to be manifested during (-)-strand synthesis.

Ded1p-driven suppression of replication of degRNAs might be critical in cells loaded with cytosolic ribonucleases that likely generate many truncated viral RNAs. These defective RNAs could likely compete with the full-length viral RNAs for viral- and host factors, thus leading to reduced viral replication. However, the co-opted cellular Ded1p helicase facilitates proper replication of TBSV RNAs and protects TBSV from competition by defective viral genomes. Since Ded1p inhibits the replication of recRNAs or degRNAs lacking the authentic 5’ end sequence through blocking the (-)-strand synthesis on these RNA templates, lesser amount of defective viral dsRNAs could accumulate. The reduced amount of dsRNA is an advantage for the virus, because dsRNAs could efficiently trigger antiviral responses, such as RNAi (or RNA silencing in plants) [72–74].

### The co-opted eIF4AIII-like AtRH2 helicase facilitates viral RNA recombination and promotes the replication of the 5’-truncated viral RNAs

Another surprising finding in this study is the stimulatory effect of a second group of co-opted cellular DEAD-box helicases on TBSV RNA recombination. Accordingly, over-expression of the eIF4AIII-like AtRH2 in yeast or plant cells led to increased level of recRNA accumulation (Figs. 7–8).

The AtRH2 helicase binds to the 5’ proximal region of the viral (-)RNA, which harbors the RIII(-) REN, resulting in localized unwinding of the dsRNA replication intermediate [50,54]. Although this unwinding process is important for the replication of the full-length TBSV RNA, it seems that it only works “properly” for TBSV replication when Ded1p/AtRH20 helicase is also present in the replicase complex. Based on these observations, the emerging concept is that the coordinated action of these two co-opted cellular helicases is required for efficient replication of the full-length viral RNA. If Ded1p is missing or the eIF4AIII-like AtRH2 is present in excess amount within the replicase complex, then the frequency of viral RNA recombination increases and replication of 5’-truncated viral degRNAs becomes more efficient. Therefore, these conditions favor the rapid evolution of TBSV, which could be advantageous under some circumstances, but disadvantageous when the wt TBSV is the best-adapted to the host/environment.

### Model for the co-opted cellular helicases in TBSV replication, viral RNA recombination and maintenance of viral genome integrity

Previous works revealed roles for Ded1p/AtRH20 and AtRH2 DEAD-box helicases during TBSV (+)-strand synthesis [49,54], which was based on local unwinding of the dsRNA replication intermediate to facilitate initiation of (+)-strand synthesis by the viral replicase (Fig. 9A). However, this work unearthed a novel function for Ded1p helicase by showing an increased level of (-)RNA production from recRNAs and degRNAs in yeast expressing mutant Ded1p or with depleted level of Ded1p. To explain these findings, we propose that Ded1p helicase facilitates the displacement of the viral p92 RdRp protein from the dsRNA product at the end of (-)-strand synthesis, as shown schematically in Fig. 9A. In case of the full-length viral RNA, the localized unwinding of the “left side” of the dsRNA then promotes the association of the p92 RdRp with the 3’ *cis*-acting elements in the (-)RNA portion of dsRNA, followed by (+)-strand synthesis via strand-displacement mechanism as shown before [50]. Thus, basically, Ded1p/AtRH20 helicases could promote the switch from (-)- to (+)-strand synthesis.

In case of the 5’-truncated RNAs, Ded1p/AtRH20 helicases could likely displace the p92 RdRp from the 5’ end of the degRNAs (Fig. 9B). Displacement of p92 RdRp from the template would likely inhibit template-switching events during (-)-strand synthesis. Accordingly, *in vitro* assays support this model by providing evidence that Ded1p promotes dissociation of p92 RdRp from the viral RNA (Fig. 5). Moreover, Ded1p helicase might not be able to open the ds degRNA to facilitate initiation of (+)-strand synthesis due to the absence of RI(-) sequence (i.e. Ded1p binding sequence) in the (-)degRNA [49]. Indeed, all degRNAs identified lack the authentic 3’ end viral sequences in the (-)RNA [15,18,65]. Based on these, we propose that Ded1p helicase suppresses the use of 5’-truncated degRNAs in (+)-strand synthesis. Overall, the p92 displacement ability of Ded1p likely inhibits template-switching RNA recombination and the replication of recRNAs (Fig. 9B).

However, when Ded1p is depleted or mutant Ded1p is present, then p92 RdRp protein will not be efficiently displaced from the dsRNA [after finishing (-)RNA synthesis on the (+)RNA template], and this condition then facilitates template-switching-based RNA recombination (Fig. 9B). In addition, the replication of degRNAs and recRNAs is also increased in the absence of functional Ded1p, likely due to the presence of AtRH2 type helicase in the VRCs, which facilitates unwinding of the “right-side” of the dsRNA template, thus promoting re-initiation on the plus-strands of dsRNA templates to generate new minus-strands (Fig. 9B). AtRH2 cannot facilitate re-initiation on the (+)RNA when Ded1/AtRH20 is present due to the recruitment of p92 to the (-) 3’-end sequences by Ded1p, long-range RNA-RNA interactions and additional cellular factors, such as GAPDH, as described earlier [54]. Altogether, the above events could explain the increased level of (-)RNAs from degRNAs and recRNAs in yeast either expressing mutant Ded1p or with depleted Ded1p.

Overall, the novel function of the DDX3-like Ded1p/RH20 helicases is the down-regulation/inhibition of (-)RNA synthesis by promoting the efficient switch from (-)RNA to (+)RNA synthesis. Interestingly, this feature requires the authentic viral 3’ end sequences on the (-)RNA, suggesting similarities between telomeres and viral RNA synthesis in protection of the ends of linear nucleic acids [75,76]. Those viral RNAs lacking the authentic terminal sequences could replicate less efficiently in the presence of Ded1p/AtRH20 helicases, suggesting that TBSV recruits a cellular helicase to protect and promote the replication of the full-length viral RNAs, while suppressing the accumulation of recRNAs and degRNAs during viral infections. Therefore, based on this work, a new concept emerges on the roles of co-opted cellular helicases in maintaining viral genome integrity.

## Materials and Methods

### Yeast strains and expression plasmids

The yeast (*Saccharomyces cerevisiae*) strain BY4741 (*MAT*a *his3*(*1 leu2*(*0 met15*Δ*0 ura3*(*0*), R1158 and TET::DED1 (yTHC library) were obtained from Open Biosystems. The temperature-sensitive (ts) yeast strains ded1–95^ts^ and ded1–199^ts^ were of generous gift from C. Boone (U. Toronto).

The yeast expression plasmids LpGAD-His92 (containing CNV p92^pol^ gene behind the *CUP1* promoter, *LEU2* selection), and HpHisGBK-His33/DI-AU-FP (co-expressing p33 from the *CUP1* promoter and DI-AU-FP repRNA from *GAL1* promoter, *HIS3* selection), HpHisGBK-HFHis33/DI-72 (co-expressing p33 from the *CUP1* promoter and DI-72 repRNA from *GAL1* promoter, *HIS3* selection), UpGBK-His33/DI-AU-FP (co-expressing p33 from the *ADH1* promoter and DI-AU-FP repRNA from *GAL1* promoter, *URA3* selection) have been previously described [8]. Plasmid HpGBK-His33/DI-RIIΔ70 was made by PCR-amplification of DI-72RIIΔ70 with primers #1546 (CCGCGAATTCACGGATTAGAAGCCGCCGAGCGGGT) and #1069 (CCGGTCGAGCTCTACCAGGTAATATACCACAACGTGTGT). The PCR product was restriction digested with *EcoR*I and *Sac*I and ligated into the plasmid HpGBK-His33/DI-72 to replace the full-length DI-72 fragment.

The *E*. *coli* expression plasmids pMAL-Ded1, pMAL-D1, pMAL-D11 and pMAL-RH2 were prepared previously [49,54]. To prepare plasmids for expression of MBP-tagged Ded1p ts mutants, genomic DNA of *ded1–95* or *ded1–199* strains were used as templates in PCR with primers #3956 (CCAGCTGCAGTCACCACCAAGAAGAGTTG) and #3957 (CCAGGAATTCATGGCTGAACTGAGCGAACAAG). The PCR products were cloned into pMalc-2x vector at *EcoR*I and *Pst*I sites. For GST-Ded1p expression, pMAL-Ded1 was used as a template in PCR with primers #4308 (TGGAACTTGGAATTGTTTACACCTTAGTCTGTTGACTTAA) and #4309 (CCAGCTCGAGTCACCACCAAGAAGAGTTG). The PCR products were cloned into pGEX-his-RE vector at *Spe*I and *Xho*I site.

The plant expression plasmids pGD-RH2, pGD-RH20, pGD-CNV and pGD-DI-AU-FP were described previously [54,65]. To obtain plasmids HpGBK-His-RH20 and HpGBK-His-RH2, the plasmids pMAL-RH20 or pMAL-RH2 [54,61], respectively, were used as templates in PCR with primers #4315 (CCAGGGATCCATGAGTGCATCATGGGCAG) and #4316 (CCAGCTGCAGCTAATCCCAAGCACTGGTC) for RH20; and #4816 (CCAGGGATCCATGGCGACAGCGAATCCTGG) and #5117 (CCAGCTGCAGTTAGATAAGATCAGCTACATTC) for RH2 open reading frames. The PCR products were cloned into HpGBK-His vector at *BamH*I and *Pst*I sites.

### Yeast transformation and cultivation

Yeast strains were co-transformed with plasmids by using the lithium acetate/ssDNA/polyethylene glycol method, and transformants were selected by complementation of auxotrophic markers [77]. For TBSV recombination assay in BY4741, ded1–95^ts^, ded1–199^ts^, R1158 and TET::DED1, yeast strains were co-transformed with LpGAD-His92 and HpGBK-His33/DI-AU-FP, HpGBK-His33/DI-RIIΔ70 or HpHisGBK-HFHis33/DI-72. The transformed BY4741, ded1–95^ts^, and ded1–199^ts^ strains were pre-grown at 23°C overnight in SC-LH^-^ (synthetic complete media without histidine and leucine) media with 2% galactose. Then, 50 μM CuSO_4_ was added to the yeast cultures to launch virus replication and recombination. Yeast was grown at either 23°C or 29°C for 24 h before sample collection for analysis. The transformed R1158 and TET::DED1 strains were pre-grown at 29°C overnight in SC-ULH^-^ (synthetic complete media without uracil, histidine and leucine) media with 2% galactose containing 10 μg/ml doxycycline. Then, 50 μM CuSO_4_ was added to the yeast cultures to launch virus replication and recombination at 23°C or 29°C for 24 h.

In the complementation study, BY4741 and ded1–199^ts^ strains were co-transformed with LpGAD-His92, UpGBK-His33/DI-AU-FP and the indicated plasmids (HpGBK-HisRH20 or HpGBK-HisRH2) expressing one of the host helicases. The transformed yeast strains were pre-grown at 23°C overnight in SC-ULH^-^ media with 2% galactose, followed by the addition of 50 μM CuSO_4_ and culturing at 23°C or 29°C for 24 h.

For viral RNA stability assay, BY4741 ded1–95^ts^, and ded1–199^ts^ strains were transformed with UpYC-DI-RIIΔ70 [18]. The transformed yeast strains were grown at 23°C in SC-U^-^ media with 2% galactose. After 24 h, the cultures were re-suspended in SC-U^-^ media with 2% glucose and grown at 23°C or 29°C. The samples were collected at given time points mentioned in figure legend.

To observe the TBSV DI-(RI RNA recombination profile in BY4741, (Xrn1, (Met22, and ded1–199^ts^ yeast strains, they were co-transformed with HpGBK-His33, LpGAD-His92 and pYC2-DI-(RI. The transformed cultures were inoculated on to ULH^-^/glucose media and grown at 23°C for 12 hrs. Yeast cultures were collected by centrifugation and dissolved in ULH^-^/galactose media supplemented with 50 μM CuSO_4_. Cultures were grown at 23°C for two days before sample collection for RNA analysis.

### Tombusvirus RNA analysis

TBSV RNA recombination was analyzed using total RNA extracted from yeast and plants. Standard RNA extraction and Northern blot analysis was performed as described in previous publication [78]. To detect TBSV (+)RNA or (-)RNA, we prepared ^32^P-labeled DI-72RIII/IV probe with *in vitro* T7-based transcription using PCR-amplified DNA obtained on HpGBK-His33/Gal1-DI-72 template with primers #22 (GTAATACGACTCACTATAGGGCTGCATTTCTGCAATGTTCC)/ #1165 (AGCGAGTAAGACAGACTCTTCA) for (+)RNA detection; and primers #18 (GTAATACGACTCACTATAGGAGAAAGCGAGTAAGACAG) / #1190 (GGGCTGCATTTCTGCAATG) for (-)RNA detection.

Typhoon FLA 9500 system (GE) and ImageQuant TL software were used to detect and quantify the bands in the gels. The repRNA and degRNA bands were identified based on molecular markers, while the recRNAs were identified based on previously characterized recRNAs [15,16,58,65]. Only the bands representing the major recRNAs (which are pointed at in figures) were quantified. All these RNAs were normalized based on ribosomal RNA level in all samples.

### Recombinant protein purification from *E coli*

Recombinant MBP-tagged helicase proteins and MBP-tagged TBSV p33 and p92 replication proteins or MBP-p92Δ167N were expressed in *E coli* and purified as published before [49]. Briefly, the expression plasmids were transformed into *E*. *coli* strain BL21 (DE3) CodonPlus. Protein expression was induced by isopropyl-β-D-thiogalactopyranoside (IPTG) at 16°C for 8 h. After collection of the cultures by centrifugation at 4000 xg for 5 min, the cells were re-suspended and broken in reduced-salt column buffer (25 mM NaCl, 30 mM HEPES-KOH pH 7.4, 1 mM EDTA, 10 mM β-mercaptoethanol). The lysate was centrifuged at 14,000 rpm for 10 min to remove cell debris. Then, the supernatant was incubated with amylose resin (NEB) at 4°C for 1 h. After washing the resin with 50 ml reduced-salt column buffer (without β-mercaptoethanol), the recombinant proteins were eluted in maltose buffer (column buffer containing 0.18% (W/V) maltose).

### *In vitro* replication assay using yeast membrane-enriched fractions

The membrane-enriched fraction (MEF) was obtained as published previously [62,78]. Briefly, yeast strains were transformed and grown as described above for TBSV recombination in yeast. Yeast cultures were collected and processed to obtain the MEFs containing the *in vivo*-assembled replicase complexes as previously described [78]. Each membrane fraction preparation was adjusted based on the relative amounts of His_6_-tagged p33 and comparable amounts of replicase (based on p33) from each preparation were used in the subsequent *in vitro* replicase assay. The replicase assay was performed as described [62,78]. Briefly, the *in vitro* assay (50 μl) contained 10 μl of the normalized MEF preparations, 10 mM DTT, 50 mM Tris-Cl pH 8.0, 10 mM MgCl_2_, 0.1 U RNase inhibitor, 1 mM ATP, 1 mM CTP, 1 mM GTP and 0.1 μl of α^32^P-UTP (3000 Ci/mmol). Reaction mixtures were incubated at 25°C for 3h, followed by phenol/chloroform extraction and isopropanol/ammonium acetate (10:1) precipitation. ^32^P-labeled RNA products were analyzed in 5% acrylamide/8 M urea gels. To detect the membrane associated RNA, membrane preparations that contained comparable amounts of replicase were used to extract the viral RNA by standard phenol/chloroform extraction and isopropanol/ammonium acetate (10:1) precipitation. Then, the RNAs were analyzed by Northern blotting with (+) or (-) RNA specific probes.

### Yeast cell free extract (CFE) based *in vitro* replication assay

CFEs from BY4741 and TET::DED1 treated with 10 μg/ml doxycycline were prepared as described earlier [29,62] and adjusted to contain comparable amounts of total protein. The in vitro CFE-based assays were performed in 20 μl total volume containing 1 μl of adjusted CFE, 0.5 μg DI-72 (+), DI-RIIΔ70 (+) or DI-AU-FP (+)RNA transcripts (separately), 0.5 μg purified MBP-p33, 0.5 μg purified MBP-p92pol (both recombinant proteins were purified from E. coli) [79], 30 mM HEPES-KOH, pH 7.4, 150 mM potassium acetate, 5 mM magnesium acetate, 0.13 M sorbitol, 0.4 μl actinomycin D (5 mg/ml), 2 μl of 150 mM creatine phosphate, 0.2 μl of 10 mg/ml creatine kinase, 0.2 μl of RNase inhibitor, 0.2 μl of 1 M dithiothreitol (DTT), 2 μl of 10 mM ATP, CTP, and GTP and 0.25 mM UTP and 0.1 μl of 32P-UTP. 0.3 μg of MBP-Ded1, MBP-D1 or MBP-D11, respectively, was added to the assay to test their activities during viral RNA synthesis. Reaction mixtures were incubated for 3 h at 25°C, followed by phenol/chloroform extraction and isopropanol/ammonium acetate (10:1) precipitation. ^32^P-labeled RNA products were analyzed in 5% acrylamide/8 M urea gels [62].

### Gel mobility shift assay

The ^32^P-labeled full-length DI-72 (-)RNA and the RI(+) RNA were generated as described [79]. Ded1p and ts mutants were incubated with 5 ng of ^32^P-labeled DI-72(-) RNA probe in a binding buffer (50 mM Tris-HCl [pH 8.2], 10 mM MgCl_2,_ 1 mM EDTA, 10% glycerol, 200 ng of yeast tRNA [Sigma], and 2 U of RNase inhibitor [Ambion]) at 25°C for 15 min. After the binding, the samples were analyzed by 5% nondenaturing PAGE performed at 200 V for 1 h in a cold room. To test the template release activity, briefly, ^32^P-labeled RI(+)RNA probe was incubated with p92-Δ167N at 25°C for 15 min, followed by adding affinity-purified GST-Ded1p to the reaction with or without 1 mM ATP, then the reaction was incubated at 25°C for 30 min. In addition, probe was also incubated with proteins in a different order mentioned in figure legend.

### Biotinylation and template release assay

First, p92-Δ167N RdRp assay was used to produce the biotin-labeled partial dsRNA product [21]. Briefly, the *in vitro* RdRp reaction was performed in 20 μl total volume containing 1 μl of adjusted CFE (soluble fraction only), 0.5 μg DI-mini (+)RNA transcript [21], 0.5 μg affinity-purified MBP-p92-Δ167N, 30 mM HEPES-KOH, pH 7.4, 150 mM potassium acetate, 5 mM magnesium acetate, 0.13 M sorbitol, 0.2 μl actinomycin D (5 mg/ml), 2 μl of 150 mM creatine phosphate, 0.2 μl of 10 mg/ml creatine kinase, 0.2 μl of RNase inhibitor, 0.2 μl of 1 M dithiothreitol (DTT), 2 μl of 10 mM ATP, CTP, and GTP and 0.1 mM UTP and 0.1 μl of biotin-UTP. Reaction mixture was incubated at 25°C for 30 min. Note that we combined 10 separate *in vitro* reactions in the subsequent experiment. After incubation, the free, unincorporated biotin-UTP was removed by Sepharose G-25 column. Then, 200 μl of *in vitro* RdRp reaction mixture were incubated with Strepavidin-beads (MagneSphere Magnetic Separation Products, Promega) at 25°C for 10 min to capture the biotin-labeled RNA and the RNA-bound p92-Δ167N RdRp as well. Then, we washed the beads once with 0.1% SSC buffer, followed by incubation of the beads with GST-Ded1p or GST (as a control) in RdRp buffer (10 mM DTT, 50 mM Tris–Cl pH 8.0, 10 mM MgCl_2_) with 1 mM ATP at 25°C for 10 min to elute (release) the p92-Δ167N RdRp from the streptavidin-bound RNA. Then, we collected and precipitated the eluted proteins with 10% TCA. The precipitated proteins (eluate fraction in Fig. 5C) were dissolved in 30 μl SDS buffer. We also recovered the p92-Δ167N RdRp from the streptavidin-bound RNA by boiling the beads in 30 μl SDS buffer for 5 min (SDS fraction in Fig. 5C). All the protein samples were analyzed by Western blotting method with anti-MBP antibody to detect the amount of p92-Δ167N RdRp in the obtained samples.

### Tombusvirus recombination in *Nicotiana benthamiana*

Cultures of *Agrobacterium tumefaciens* C58C1 strain carrying pGD-RH2 or pGD-RH20 were used for transient expression of *Arabidopsis thaliana* RH2 and RH20 [54,61]. *N*. *benthamiana* plants were infiltrated with *A*. *tumefaciens* carrying pGD-RH2 (OD_600_ = 0.3) or pGD-RH20 (OD_600_ = 0.3) together with pGD-CNV (OD_600_ = 0.3) and pGD-DI-AU-FP (OD_600_ = 0.3) to launch tombusvirus replication and induce RNA recombination. Leaf infiltration with *A*. *tumefaciens* carrying “empty” pGD plasmid was used as a control. We also performed agroinfiltration with pGD-p33 + pGD-p92+ pGD-DI-ΔRI at a final concentration of 0.3 (OD_600_). Three and four days after agro-infiltration, samples from the agro-infiltrated leaves were collected from the agroinfiltrated leaves. Total RNA was extracted and Northern blot analysis was performed as previously described [16].
